# Managing the complexity of communication: regulation of gap junctions by post-translational modification

**DOI:** 10.3389/fphar.2013.00130

**Published:** 2013-10-22

**Authors:** Lene N. Axelsen, Kirstine Calloe, Niels-Henrik Holstein-Rathlou, Morten S. Nielsen

**Affiliations:** ^1^Department of Biomedical Sciences and The Danish National Research Foundation Centre for Cardiac Arrhythmia, Faculty of Health and Medical Sciences, University of CopenhagenCopenhagen, Denmark; ^2^Department of Veterinary Clinical and Animal Sciences and The Danish National Research Foundation Centre for Cardiac Arrhythmia, Faculty of Health and Medical Sciences, University of CopenhagenCopenhagen, Denmark

**Keywords:** connexin, post translational modification, phosphorylation, sumoylation, nitrosylation, methylation, acetylation, ubiquitination

## Abstract

Gap junctions are comprised of connexins that form cell-to-cell channels which couple neighboring cells to accommodate the exchange of information. The need for communication does, however, change over time and therefore must be tightly controlled. Although the regulation of connexin protein expression by transcription and translation is of great importance, the trafficking, channel activity and degradation are also under tight control. The function of connexins can be regulated by several post translational modifications, which affect numerous parameters; including number of channels, open probability, single channel conductance or selectivity. The most extensively investigated post translational modifications are phosphorylations, which have been documented in all mammalian connexins. Besides phosphorylations, some connexins are known to be ubiquitinated, SUMOylated, nitrosylated, hydroxylated, acetylated, methylated, and γ-carboxyglutamated. The aim of the present review is to summarize our current knowledge of post translational regulation of the connexin family of proteins.

## INTRODUCTION

**FIGURE 1:**
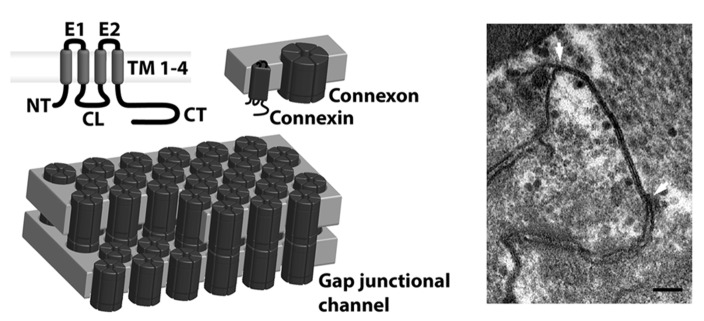
**Connexin structure**. Gap junctional channels are comprised of connexins, which have four transmembrane domains (TM1-4), two extracellular loops (E1-2), a cytoplasmic loop (CL), N-terminus (NT) and C-terminus (CT; upper left panel). Six connexins oligomerize and form connexons (upper middle panel), which are transported to the plasmamembrane. Here they dock with a connexon from a neighboring cell and form full intercellular gap junctional channels (lower left panel), that upon opening mediate exchange of ions and small metabolites. When many channels aggregate, they form a gap junctional plaque, which can be seen as a pentalaminar structure in the transmission electron microscope. A plaque between two rat ventricular myocytes can be seen between the arrows in the right panel of the figure (scale bar 100 nm). The image was obtained in collaboration with Klaus Qvortrup, Core Facility for Integrated Microscopy, University of Copenhagen.

Connexins are a family of membrane proteins forming gap junctional channels. Each connexin is a four membrane spanning protein with two extracellular loops, an intracellular loop and intracellular N- and C-termini (see **Figure 1**). Six connexins oligomerize to form a connexon, which, once inserted in the plasma membrane, may dock with a connexon from a neighboring cell and form an intercellular gap junctional channel. When many intercellular channels aggregate, they form a gap junction plaque, which present a typical penta-laminar structure when viewed in transmission electron microscope images (**Figure 1**, right panel). In some cases, free connexons may open directly to the extracellular medium in which case they are often referred to as hemichannels. This review will primarily deal with intercellular gap junctional channels.

Gap junctions are responsible for direct intercellular communication in vertebrates and the connexin family has 21 members in humans and 20 in mice (Willecke et al., 2002). The connexins are named after their approximate molecular weight (e.g., Cx43 is a protein of app. 43 kDa), whereas the genes encoding them are named GJX followed by a number with X indicating group (α, β, γ, δ or ε) based on homology and length of their cytoplasmic loop (for more information about nomenclature, see Nielsen et al., 2012). A list of gene and protein names for human and mouse connexins is given in **Table 1**. In the present review, we use the name of the human ortholog in general but may also state species dependent names where relevant to avoid confusion.

**Table 1:** Nomenclature of human and mouse connexin genes and proteins.

Human Gene name	Human Protein name	Mouse gene name	Mouse protein name
GJE1	hCx23	Gje1	mCx23
GJB7	hCx25	No mouse ortholog	No mouse ortholog
GJB2	hCx26	Gjb2	mCx26
GJC3	hCx30.2	Gjc3	mCx29
GJB6	hCx30	Gjb6	mCx30
GJB4	hCx30.3	Gjb4	mCx30.3
GJB3	hCx31	Gjb3	mCx31
GJB5	hCx31.1	Gjb5	mCx31.1
GJD3	hCx31.9	Gjd3	mCx30.2
GJB1	hCx32	Gjb1	mCx32
No human ortholog	No human ortholog	Gja6	mCx33
GJD2	hCx36	Gjd2	mCx36
GJA4	hCx37	Gja4	mCx37
GJA5	hCx40	Gja5	mCx40
GJD4	hCx40.1	Gjd4	mCx39
GJA1	hCx43	Gja1	mCx43
GJC1	hCx45	Gjc1	mCx45
GJA3	hCx46	Gja3	mCx46
GJC2	hCx47	Gjc2	mCx47
GJA8	hCx50	Gja8	mCx50
GJA9^*^	hCx59	No mouse ortholog	No mouse ortholog
GJA10	hCx62	Gja10	mCx57

* Previously named GJA10

Gap junctions are instrumental in many physiological phenomena, from embryonic development to propagation of cardiac action potentials. Giving the diversity of these tasks, it is not surprising that the connexins, which form the gap junctional channels, come in many isoforms. By the expression of different connexins, cells can regulate coupling in a tissue and time dependent manner.

The connexin isoforms differ in their permeability to larger molecules, termed metabolic coupling; and current in the form of atomic ions, termed electrical coupling. Although it is often stated that gap junctional channels are permeable to substances up to 1 kDa, metabolic coupling is highly variable among isoforms and permeability is often unpredictable with respect to permeant size and charge. Most of our current knowledge on metabolic coupling comes from studies of fluorescent tracer molecules and some caution is needed when comparing studies using different tracers, also it should be kept in mind that permeability to biologically relevant molecules cannot necessarily be inferred from tracer permeability. More information about permeability of connexin channels can be found in (Harris and Locke, 2009; Nielsen et al., 2012). Electrical coupling can be measured by dual patch clamp and investigated as either the total conductance between cells in a pair, termed macroscopic coupling, or single channel conductance, which is usually determined under partial uncoupling by agents like octanol.

The characteristics of the various connexin isoforms enable them to fulfill different physiological roles that may be more or less connexin specific. In the case of the development of cardiac morphology, Cx43 may be exchanged with Cx40 without consequence and is thus relatively unspecific; however, the same exchange renders homozygous females unable to wean their pups and males infertile, which shows that these processes are high dependent on the specific properties of Cx43 (Plum et al., 2000).

Despite the possibility of altering intercellular coupling by changing the connexin expression profile, cells often need to regulate communication acutely by altering the function and localization of the connexins at hand. This is often mediated by post translational modifications (PTMs). The most widely investigated PTMs in connexins are phosphorylations and ubiquitinations, but in recent years studies have shown how other PTM such as ubiquitination, SUMOylation, nitrosylation, hydroxylation, acetylation, methylation, and γ-carboxyglutamation also play important roles in the regulation of intercellular communication.

Intercellular coupling is determined by both the number of channels incorporated in gap junctions, their activity and selectivity. The number of channels is regulated by transcription, translation, oligomerization, trafficking to and from the membrane, and degradation. Pre-translational events (transcription and translation) does of course not involve PTM (for review of these processes in connexins, see Nielsen et al., 2012), but PTMs are involved in the regulation of all other aspects of the connexin lifecycle. The activity of gap junctional channels is determined by their open probability, conductance and selectivity, each of which can be regulated by PTMs. In the following, we will review the state of knowledge of how various PTMs regulate connexin function.

## PHOSPHORYLATION – A KEY PLAYER IN THE REGULATION OF GAP JUNCTIONS

The covalent binding of phosphate groups to either serine, threonine or tyrosine residues of proteins is termed protein phosphorylation. The addition of a phosphate group to a protein is facilitated by various protein kinases, whereas removal of a phosphate group, dephosphorylation, is mediated by protein phosphatases. Protein phosphorylation was first described by Edmond Fischer and Edwin Krebs, who were awarded the Nobel Prize for *“their discoveries concerning reversible protein phosphorylation as a biological regulatory mechanism” *in 1992*.* Today, phosphorylation and dephosphorylation is well described and potentially the most common way of controlling the activity and function of proteins in biological systems. Following translation of a protein, the phosphorylation state of the protein usually determine the three-dimensional folding and conformation, the intracellular trafficking and activity of the protein, as well as its interaction with other proteins. Phosphorylation of proteins is therefore a key player in the regulation of all forms of cellular processes. 

The first evidence that connexins are phosphoproteins was published in the 1980s (Saez et al., 1986; Takeda et al., 1987). Since then, tremendous amounts of work have contributed to our current knowledge of site-specific phosphorylation and dephosphorylation and its contribution to the post-translational regulation of connexins. All the connexin family members are now known to be phosphoproteins and connexin phosphorylation/dephosphorylation is involved in all stages of the connexin life-cycle, the regulation of electrical and metabolic coupling of gap junction channels, as well as regulation of connexin interaction with other proteins. The phosphorylation state of connexins is dependent on interplay between various kinases and phosphatases, it is often cell- or tissue-type specific and it is further affected by various physiological and pathological conditions. This part of the review aims at summarizing the current knowledge of connexin phosphorylation, while highlighting the contradictions that exist and turning attention to the areas, which need further elucidation.

### PHOSPHORYLATION OF CONNEXIN43

Of the 21 identified members of the connexin family, the 43 kDa subtype, connexin 43 (Cx43) is not only the most widely expressed in mammalian cells, it is also the most intensively studied connexin. Cx43 is translated as a 40 kDa protein, which becomes phosphorylated to a 41 kDa form soon after synthesis (Puranam et al., 1993). The early phosphorylation of Cx43, which results in the 41 kDa form, occurs in the ER or *cis-medial *Golgi (Puranam et al., 1993). A more extensive phosphorylation, however, takes place later in the secretory pathway or following formation of gap junction channels at the plasma membrane. Today, 21 phosphorylation sites are described for Cx43 and a substantial amount of experimental work have demonstrated that all stages of the Cx43 “life cycle” are modified by post-translational phosphorylation of Cx43. In addition, pathophysiological conditions, such as ischemia (Beardslee et al., 2000; Axelsen et al., 2006), hemodynamic volume overload (Rucker-Martin et al., 2006; Qu et al., 2009) and diabetes (Lin et al., 2006; Howarth et al., 2008), may affect Cx43 phosphorylation and thereby gap junction coupling between Cx43 expressing cells.

### SITE SPECIFIC CX43 PHOSPHORYLATION AND THE RESPONSIBLE PROTEIN KINASES

**FIGURE 2:**
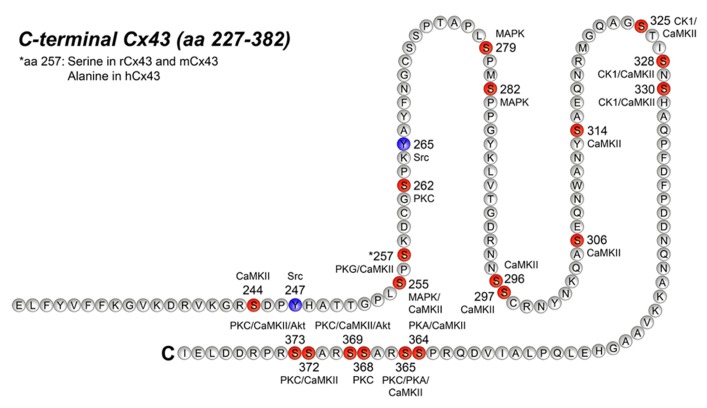
**Phosphorylation sites in the C-terminal tail of Cx43**. This figure represents the C-terminal tail of Connexin43 (Cx43; amino acid, aa 227–382) including its phosphorylation sites and its respective kinases. Serine (S) phosphorylation sites are presented in red and tyrosine (Y) phosphorylation sites are presented in purple. CaMKII, Ca2+/calmodulin-dependent kinase II; PKG, protein kinase G; PKC, protein kinase C, MAPK, mitogen-activated protein kinase; CK1, casein kinase 1; PKA, protein kinase A. For references see text and **Table 2**.

**Figure 2** illustrates all the specific phosphorylation sites identified in Cx43, as well as the specific kinases that facilitate their phosphorylation. In addition to the sites and kinases shown in **Figure 2**, bioinformatics has identified additional phosphorylation consensus sites in Cx43 and heat map analysis reveal that the majority of the phosphorylation sites may be recognized by multiple kinases (reviewed by Chen et al., 2013). In the following, we will focus on the specific phospho-sites and kinases shown in **Figure 2**, since they are all verified by biological experiments.

**Table 2:** Connexin 43 post translational modification (PTM) sites including kinases responsible for phosphorylation (P).

Residue	PTM (Kinase(s))	Reference
K114	SUMOylation	Kjenseth etal. (2012)
K234	SUMOylation	Kjenseth etal. (2012)
S244	P (CaMKII)	Huang etal. (2010)
Y247	P (Src)	Lin etal. (2001), Solan and Lampe (2007)
S255	P (MAPK/CaMKII)	Warn-Cramer etal. (1996), Cameron etal. (2003), Huang etal. (2010)
S257^*^	P (PKG/CaMKII)	Kwak etal. (1995a), Kwak and Jongsma (1996), Huang etal. (2010)
S262	P (PKCε)	Doble etal. (2004), Axelsen etal. (2006), Srisakuldee etal. (2009)
Y265	P (Src)	Giepmans etal. (2001), Lin etal. (2001), Toyofuku etal. (2001), Solan and Lampe (2007)
C271	Nitrosylation	Straub etal. (2011)
S279	P (MAPK)	Warn-Cramer etal. (1996)
S282	P (MAPK)	Warn-Cramer etal. (1996)
S296	P (CaMKII)	Axelsen etal. (2006), Huang etal. (2010)
S297	P (CaMKII)	Axelsen etal. (2006), Huang etal. (2010)
S306	P (CaMKII)	Axelsen etal. (2006), Huang etal. (2010)
S314	P (CaMKII)	Huang etal. (2010)
S325	P (CK1/CaMKII)	Cooper and Lampe (2002), Huang etal. (2010)
S328	P (CK1/CaMKII)	Cooper and Lampe (2002), Huang etal. (2010)
S330	P (CK1/CaMKII)	Cooper and Lampe (2002), Huang etal. (2010)
364	P (PKA/CaMKII)	TenBroek etal. (2001), Shah etal. (2002), Huang etal. (2010)
S365	P (PKC/PKA/CaMKII)	Shah etal. (2002), Huang etal. (2010)
S368	P (PKC)	Saez etal. (1997), Lampe etal. (2000), Shah etal. (2002), Bao etal. (2004), Axelsen etal. (2006), Ek-Vitorin etal. (2006)
S369	P (PKC/Akt/CaMKII)	Shah etal. (2002), Park etal. (2007), Huang etal. (2010)
S372	P (PKC/CaMKII)	Saez etal. (1997), Shah etal. (2002), Huang etal. (2010)
S372	P (PKC/Akt/CaMKII)	Shah etal. (2002), Park etal. (2007), Huang etal. (2010)

* Amino acid residue 257 of rat Cx43 is a serine. In human Cx43, residue 257 is an alanine. CaMKII, Ca^2^^+^/calmodulin-dependent kinase II; MAPK, mitogen-activated protein kinase; PKG, protein kinase G; PKC, protein kinase C; CK1, casein kinase 1; PKA, protein kinase A. Akt is also known as protein kinase B.

### Ser244 AND Ser314

Ser244 and Ser314 were recently identified as potential phosphorylation sites in Cx43. Using high-resolution mass spectrometry and a peptide containing the full length of the C-terminal tail of Cx43, Huang et al. (2010) showed that Ser244 and Ser314, along with 13 other serine sites (see **Table 2**), are targets for *in vitro* phosphorylation by Ca^2^^+^/calmoduline-dependent kinase II (CaMKII). CaMKII is involved in a variety of cellular processes, such as Ca^2^^+^ homeostasis, transcription and apoptosis (reviewed by Braun and Schulman, 1995), however, the specific role of CaMKII in the regulation of Cx43, and whether phosphorylation of Ser244 and Ser314 plays a role *in vivo* remains to be investigated.

### Tyr247 AND Tyr265

Tyr247 and Tyr265 are the only 2 out of the 21 described phosphorylation sites in Cx43, which are tyrosine sites, and both sites are targets of the oncoprotein and tyrosine Src kinase. Activation of c-Src causes direct phosphorylation of Tyr265, which leads to reduced electrical conductance in the absence of changes in the amount of Cx43 gap junction channels (Postma et al., 1998; Giepmans et al., 2001). In addition, v-Src (the constitutively active Src kinase isoform) is reported to co-localize with Cx43 present in the plasma membrane (Loo et al., 1999), an interaction, which depends on the Src-homology-2 (SH2) and SH3 domains in v-Src, as well as the Pro274-Pro284 region and phosphorylation of Tyr265 in the cytoplasmic tail of Cx43 (Kanemitsu et al., 1997). A study on Cx43 mutants has implied that phosphorylation of Tyr265 alone is not sufficient for complete gap junction closure. Instead, phosphorylation of both Tyr265 and Tyr247 are required to disrupt metabolic coupling through Cx43 gap junction channels (Lin et al., 2001). Together, these results support a model for v-Src induced Cx43 tyrosine phosphorylation, where interaction is initiated by binding of the v-Src SH3 domain to the Pro274-Pro284 region of Cx43. This interaction facilitates the phosphorylation of Tyr265, which then acts as a binding site for the SH2 domain. Binding of the v-Src SH2 domain to P-Tyr265 then strengthen the interaction between Cx43 and v-Src and facilitates the subsequent phosphorylation of Tyr247 and closure of the Cx43 gap junction channel (Lin et al., 2001). This mechanism for direct Src induced gap junction closure seems reasonable and experimentally supported. Nevertheless, a study by Zhou et al. (1999) showed that substitution of Tyr265 and/or Tyr247 with phenylalanine (which mimics a constitutive dephosphorylated tyrosine) did not interfere with the ability of v-Src to cause electrical uncoupling of gap junction channels. Instead, their study implied that phosphorylation of the mitogen-activated protein kinase (MAP kinase) sites Ser255, Ser279, and Ser282 (which are further described below) is essential for v-Src induced regulation of electrical coupling. In addition, a recent study found that Akt activation is crucial to disrupt dye transfer in v-Src transformed cells, but inhibition of Akt only recovered the metabolic coupling to 60% (Ito et al., 2010). Since Akt may cause phosphorylation of Ser369 and Ser373 in Cx43 (Park et al., 2007; for details see section on Ser364, Ser365, Ser368, Ser369, Ser372, and Ser373), this indicates that Src kinases controls several intracellular signaling pathways, and thereby affects the phosphorylation of both serine and tyrosine residues in the C-terminal tail of Cx43 with different effects on electrical and metabolic coupling. Together, these studies outlines that the control of gap junction coupling by Src kinases is extremely complex; It depends of a combination of direct phosphorylation of both Tyr247 and Tyr265, as well as interplay with several intracellular signaling pathways controlling Cx43 serine phosphorylation.

In addition to the control of gap junction channels, Src may also regulate gap junction localization. Binding of Src to Cx43 and phosphorylation of Tyr265 inhibits the endogenous interaction between Cx43 and the cytoskeleton protein zonula occludens 1 (ZO-1) in cardiomyocytes (Toyofuku et al., 2001). ZO-1 is important for the localization of Cx43 channels at the intercalated discs of the heart, and therefore, an interruption of the Cx43-ZO-1 interaction may reduce the amount of Cx43 present at the cell surface. This is further supported by Duffy et al. (2004) who found that intracellular acidification in astrocytes causes Cx43 internalization accompanied by decreased binding of ZO-1 and increased binding of Src to Cx43.

Based on the complex nature of Src kinase regulation of Cx43 gap junction channels, it is not surprising that Src induced alterations in gap junction coupling is found in several pathological conditions. Since gap junction communication is essential for controlled cell growth and cell differentiation, Src kinase induced Cx43 phopshorylation seems to play an essential role in tumorigenesis (reviewed by Pahujaa et al., 2007). Furthermore, tyrosine phosphorylation of Cx43 is suggested to play a role in cardiac arrhythmias caused by either increased activation of the renin-angiotensin-aldosterone system or metabolic inhibition. More specifically, it was shown that treatment with a c-Src inhibitor interrupts Angiotensin II mediated loss of Cx43 gap junction channels and reduces the risk of ventricular tachycardia in mice with over expression of angiotensin-converting-enzyme (ACE; Sovari et al., 2011). Furthermore, metabolic inhibition of cultured neonatal cardiomyocytes also induces tyrosine phosphorylation of Cx43 and increased association between c-Src and Cx43 (Chung et al., 2007, 2009), which indicates a role for tyrosine phosphorylation in gap junction uncoupling and/or remodeling during myocardial ischemia.

### Ser255, Ser279, AND Ser282

As indicated above, Ser255, Ser279, and Ser282 are all targets for MAP kinase phosphorylation (Warn-Cramer et al., 1996; Cameron et al., 2003). In HeLa cells, Cx43 phosphorylated at Ser255, Ser279, and Ser282 is transported correctly to gap junctional plaques, but phosphorylation of Ser279 and/or Ser282 impairs channel function and decreases both electrical and metabolic coupling (Warn-Cramer et al., 1998). In addition, a recent study demonstrates that Ser279 and Ser282 are involved in the regulation of gap junction plaque size in human pancreatic cancer cells; Johnson et al. (2013) found that phosphorylation of Ser279 and Ser282 disrupts gap junction plaque growth by triggering calthrin-mediated endocytosis of Cx43. Based upon these results, is seems that MAP kinase controlled phosphorylation of Ser255 and especially Ser279 and Ser282 regulate both electric and metabolic coupling of gap junction channels, as well as gap junction plaque size by controlling the rate of Cx43 endocytosis. However, the effects of MAP kinase induced Cx43 phosphorylation maybe cell type specific and further studies are needed to determine the specific effects *in vivo*.

### Ser257

Amino acid residue 257 of Cx43 is a serine in rats, whereas it is an alanine in the human genome. Since alanine mimics a constitutive dephosphorylated form of a serine, it could be assumed that Ser257 is not a target for phosphorylation in the rat. Nevertheless, Kwak et al. (1995a) found that activation of PKG by 8-bromoguanosine 3′:5′-cyclicmonophosphate (8Br-cGMP) increase the incorporation of P^32^ into rat Cx43 but not human Cx43 expressed in SKHep1 cells. Furthermore, phosphorylation of Cx43 by protein kinase G (PKG) was associated with a decreased macroscopic and single channel conductance in SKHep1 cells (Kwak et al., 1995a). Kwak and Jongsma (1996) also showed that PKG causes decreased macroscopic – and single channel conductance in cardiomyocytes from rats. These data shows that electrical coupling is regulated by PKG induced phosphorylation of Ser257 in the rat, whereas PKG is not involved in the regulation of Cx43 in humans.

### Ser262

Fibroblast growth factor 2 (FGF2) induced stimulation of protein kinase C (PKC; subtype ε) in cultured cardiomyocytes induces Ser262 phosphorylation along with increased cardiomyocyte proliferation (Doble et al., 2004). The same study also found that Cx43 overexpression is associated with decreased DNA synthesis and thereby decreased cell proliferation. In addition, the effect of Cx43 overexpression was inhibited when Ser262 was exchanged with aspartate that mimics a constitutive phosphorylation. These findings support a central role for Ser262 phosphorylation in Cx43 mediated control of cell proliferation.

Ser262 phosphorylation may also play a role in cardio protection against ischemic injuries and arrhythmias. Both FGF2 and ischemic preconditioning causes increased phosphorylation of Ser262, as well as protection against ischemic injury in isolated rat hearts (Srisakuldee et al., 2009). Furthermore, expression of a Cx43 mutant, where Ser262 is exchanged with alanine, exacerbate injury and death of cardiomyocytes following simulated ischemia *in vitro *(Srisakuldee et al., 2009). In addition, it was recently shown that Ser262 phosphorylation is essential for Cx43 interaction with the ATP sensitive K^+^ channel Kir6.1 in NIH3T3 cells (Waza et al., 2012). Together these data supports a role for Ser262 phosphorylation in ischemic cardio protection, potentially mediated through the interaction with Kir6.1. Nevertheless, further studies are needed to determine exactly what the physiological consequences of the Cx43-Kir6.1 interaction are.

### Ser296, Ser297, AND Ser306

That Ser296, Ser297 and Ser306 of Cx43 are subjects for phosphorylation was first identified by a mass spectrometry analysis of Cx43 purified from rat hearts (Axelsen et al., 2006). This study further showed that Ser306 is dephosphorylated within 7 min of cardiac ischemia, while Ser297 (together with Ser368, which will be discussed later in this chapter) is dephosphorylated between 15 and 30 min of ischemia, where gap junction electrical uncoupling is also known to occur (Smith et al., 1995; Beardslee et al., 2000). Furthermore, the study showed that the anti-arrhythmic peptide analog rotigaptide (which is known to prevent and convert conduction slowing during metabolic stress, Haugan et al., 2005a, b) preserves Ser297 and Ser368 phosphorylation during ischemia. The preserved phosphorylation of Ser297 and Ser368 was further correlated to a delayed onset of ischemia induced arrhythmias (Axelsen et al., 2006). In other words, these findings indicate that dephosphorylation of Ser297 and/or Ser368 is responsible for gap junction uncoupling during cardiac ischemia. Another study evaluated the effect of serine to alanine substitutions of Ser306, Ser296, and Ser297 in Cx43 transfected into HeLa cells (Procida et al., 2009). Here it was found that an alanine substitution of Ser296 and Ser297 has no significant effects on either macroscopic electrical coupling or single channel conductance. In contrast, substitution of Ser306 to alanine resulted in a 57% reduction in electrical coupling, possibly mediated by a reduction of single channel conductance. Based on these findings, it seems reasonable to conclude that several phospho-sites, including Ser297 are Ser306 are involved in the regulation of electrical coupling through Cx43 channels during ischemia or metabolic stress.

The kinase(s) responsible for phosphorylation of Ser296, Ser297, and Ser306 is still a matter of debate; While Axelsen et al. (2006) were not able to detect CaMKII induced P^32^ incorporation into a synthetic peptide containing Ser296, Ser297, and Ser306, the previously discussed mass spectrometry based study by Huang et al. (2010) did detect CaMKII induced phosphorylation on all of these specific sites. Further studies are needed to clarify these contradictory findings.

### Ser325, Ser328, AND Ser330

That Cx43 phosphorylation is involved in the regulation of trafficking, assembly and dis-assembly, as well as the localization of Cx43 gap junction channels was first suggested by Musil and Goodenough (Musil et al., 1990; Musil and Goodenough, 1991). Since then, Cooper and Lampe (2002) have shown that casein kinase 1 (CK1) induced phsophorylation of Ser325, Ser328, and Ser330 in Cx43 is a key regulatory mechanism for the formation of gap junctions channels. Furthermore, it is indicated that these specific serine phosphorylation sites are only phosphorylated when Cx43 is located in gap junction plaques (Solan and Lampe, 2007). In further support of the importance of phosphorylation of these specific sits, substitution of Ser325, Ser328, and Ser330 to alanine (which mimics a constitutively dephosphorylated serine residue) causes decreased dye coupling, as well as delayed development of electrical coupling in mouse fibroblasts (Lampe et al., 2006).

In isolated Langendorff perfused rat hearts, Ser330 becomes phosphorylated within 7 min of global no-flow ischemia, whereas it returns to the dephosphorylated state between 30 and 45 min of ischemia (Axelsen et al., 2006). In addition, ischemia induced dislocation of Cx43 from the intercalated disks to the lateral edges of cardiomyocytes (a process known as lateralization) correlates with dephosphorylation of Ser325, Ser328, and/or Ser330 (Lampe et al., 2006).

Chronic hemodynamic overload also causes dephosphorylation and delocalization of atrial Cx43 in both rats and humans, which may be related to the development of atrial fibrillation (Rucker-Martin et al., 2006). Furthermore, in mice, such hemodynamic overload induced by aortic constriction causes a time-dependent reduction in Ser325, Ser328, Ser330 phosphorylation along with progressive loss of junctional Cx43, conduction velocity slowing and increased arrhythmogenicity (Qu et al., 2009). To further explore the physiological significance of Ser325, Ser328, and Ser330 phosphorylation, Remo et al. (2011) conducted an elegant study in mutant knock-in mice where Ser325, Ser328, and Ser330 were replaced by either phosphomimetic glutamic acids or non-phosphomimetic alanines. The introduction of glutamic acid as a pseudophosphorylation resulted in gap junctions that were resistant to gap junction remodeling associated with both acute ischemia and chronic hemodynamic overload. In contrast, the phosphorylation deficient mice where Ser325, Ser328, and Ser330 were replaced by alanines, displayed aberrant gap junction expression even at baseline and increased arrhythmic susceptibility (Remo et al., 2011). Collectively, these studies verify that the phosphorylation state of Ser325, Ser328, and/or Ser330 plays an important role in both physiological and pathological regulation of Cx43 gap junction localization and function.

### Ser364, Ser365, Ser368, Ser369, Ser372, AND Ser373

Ser364 is phosphorylated by protein kinase A (PKA; TenBroek et al., 2001; Shah et al., 2002), while Ser365 may be phosphorylated by both PKA and PKC (Shah et al., 2002). PKC is also known to phosphorylate Ser368, Ser369, Ser372, and Ser373 (Saez et al., 1997; Lampe and Lau, 2000; Shah et al., 2002; Bao et al., 2004). Even though Cx43 is a relatively poor substrate for PKA compared to PKC, it was shown that initial PKA activation accelerates subsequent PKC phosphorylation (Shah et al., 2002). The link between PKA and PKC phosphorylation is further supported by the finding that FSH-induced Cx43 phosphorylation, which is mediated by PKC, is depressed by the use of the selective PKA inhibitor H89 (Yogo et al., 2006).

Besides being a potential target for both PKA and PKC, Ser365 is proposed to be a “gatekeeper,” which controls the ability of other serine residues in Cx43 to become phosphorylated. More specifically, it was reported that phosphorylation of Ser365 causes a conformational change in the c-terminal region of Cx43, which prevents PKC induced phosphorylation of Ser368 (Solan et al., 2007). These data underline the complexity of Cx43 phosphorylation and demonstrate that the phosphorylation state of one site may influence the ability of other sites to become phosphorylated.

Along with the ability of PKC to phosphorylate Cx43 at several serine residues, activation of PKC by 12-*O*-tetradecanoyl-phorbol 13-acetate (TPA), is also known to increase cardiac macroscopic conductance along with reduced single-channel conductance (Kwak et al., 1995b; Lampe et al., 2000). This PKC effect on gap junction conductance is, however, prevented when Ser368 is exchanged with an alanine (Lampe et al., 2000). These findings suggest that PKC induced Cx43 phosphorylation, and especially phosphorylation of Ser368, affects electrical coupling and the open probability of gap junction channels.

The role of Ser368 phosphorylation during myocardial ischemia and its connection to electrical conductance is, however, subject of contradicting findings. As earlier mentioned, Axelsen et al. (2006) found that Ser368 becomes dephosphorylated during no-flow ischemia in isolated rat hearts, at a time course similar to that of electric uncoupling (Smith et al., 1995; Beardslee et al., 2000). In addition, rotigaptide, which prevents and reverse conduction slowing during metabolic stress (Haugan et al., 2005a, b) and suppresses the development of cardiac arrhythmias (Xing et al., 2005; Axelsen et al., 2006), preserved phosphorylation of Ser368 during ischemia (Axelsen et al., 2006). Based on this study, it is tempting to conclude that preservation of Ser368 phosphorylation contributes to an improved electrical coupling during cardiac ischemia. The findings by Axelsen et al. (2006) are, however, in contrast to findings by Ek-Vitorin et al. (2006). They reported that phosphorylation of Ser368 causes a reduction in gap junction conductance and that 30 min of no-flow ischemia in excised mice hearts results in increased levels of Ser368 phosphorylation, despite an overall Cx43 dephosphorylation when examined by western blotting. Currently, there are no clear explanations for these contradicting findings and further research is needed to clarify to role of Ser368 phosphorylation during pathological conditions.

In addition to the regulatory role of Ser368 with respect to electrical coupling, the Cx43 PKC phosphorylation sites are also involved in the regulation of metabolic coupling. In HeLa cells, it was found that alanine substitutions of Ser365, Ser368, Ser369, and Ser373 all at once, cause a marked drop in gap junction dye transfer (Yogo et al., 2002). This effect on metabolic coupling was, however, blunted if the alanine substitution was restricted to Ser368. These findings suggest that while dephosphorylation of Ser368 alone seems sufficient to alter the electrical coupling, the metabolic coupling appears to depend on several of the specific PKC sites. In contrast to the findings in HeLa cells, where reduced metabolic coupling is related to Cx43 dephosphorylation, PKC mediated increases in Cx43 phosphorylation reduces metabolic coupling in both fibroblasts (Lampe et al., 2000) and neonatal cardiomyocytes (Kwak et al., 1995b). The reason for these contradicting findings on the connection between metabolic coupling and the phosphorylation state of Cx43 is currently unknown. It could, however, simply be a matter of the different cell-systems used and further studies are needed in order to determine the effect *in vivo*.

As described above, phosphorylation of Ser369 and Ser373 can be mediated by PKC. Both these sits are, however, also subject to phosphorylation by Akt kinase (also known as protein kinase B) (Park et al., 2007). Akt induced phosphorylation of Ser369 and Ser373 facilitates an interaction between Cx43 and 14-3-3, which plays a role in trafficking of Cx43 multimers and/or their incorporation into gap junction plaques (Park et al., 2007).

Finally, the previously discussed mass spectrometry study by Huang et al. (2010) also identified Ser364, Ser365, Ser369, Ser372, and Ser373 as CaMKII targets *in vitro*. However, it is still unknown whether CaMKII phosphorylates Cx43 *in vivo* and the potential physiological role of CaMKII induced Cx43 phosphorylation remains to be established.

### THE ROLE OF PROTEIN PHOSPHATASES

Most experimental studies have focused on the protein kinases, which are responsible for Cx43 phosphorylation. Nevertheless, regulation of Cx43 phosphorylation is not only dependent on the kinases, but also on the equilibrium between protein phosphatase and kinase activity. Even so, experimental data regarding the phosphatases that dephosphorylate Cx43 remain limited.

Under normal physiological conditions, both protein phosphatase 1 (PP1) and protein phosphatase 2A (PP2A) co-localize with Cx43 in rabbit hearts (Ai and Pogwizd, 2005), indicating a physiological role for these enzymes in the regulation of Cx43. The role of these endogenous protein phosphatases on Cx43 gap junction uncoupling during ischemia or ATP-depletion have further been evaluated in neonatal rat cardiomyocytes, adult rat cardiomyocytes, as well as isolated rat hearts; in neonatal cardiomyocytes, selective PP1 inhibitors postpone electrical uncoupling of gap junctions during ATP-depletion (Duthe et al., 2001). At the same time, addition of a specific PP1 stimulator facilitated a gradual decrease in electrical coupling, even in the presence of ATP (Duthe et al., 2001). Likewise, PP1 inhibitors decreased Cx43 dephosphorylation during ischemia in both isolated perfused rat hearts and adult cardiomyocytes (Jeyaraman et al., 2003). This study also found that treatment with the selective PP2A inhibitor fostriecin did not prevent Cx43 dephosphorylation during ischemic conditions. This indicates that PP1 is the key player in Cx43 dephosphorylation during ischemia in the rat.

Both PP1 and PP2A are also expressed in mini pig hearts, but in contrast to what was found in rabbit hearts, only PP2A co-localizes with Cx43 in the mini pig (Totzeck et al., 2008). Furthermore, when isolated mini pig hearts were exposed to 90 min of low flow ischemia, it resulted in Cx43 dephosphorylation along with an increase in total PP2A levels. The amount of Cx43-PP2A co-precipitation was, however, not affected during ischemia and ischemic pre-conditioning preserved Cx43 phosphorylation, without any effect on either PP2A levels or activity (Totzeck et al., 2008). In contrast to the findings during cardiac ischemia, another study have reported a >2.5-fold increase in the amount of PP2A, which co-localizes with Cx43 in a rabbit model of non-ischemic heart failure (Ai and Pogwizd, 2005). The increase in PP2A-Cx43 co-localization was further associated with increased levels of dephosphorylated Cx43 and decreased metabolic coupling, which was prevented in the presence of okadaic acid in a concentration, which should only inhibit PP2A. The same study also examined the amount of co-localized Cx43 and PP1, before and after long-term aortic constriction, but here they found no changes (Ai and Pogwizd, 2005). Together, these studies show that both PP1 and PP2A may affect Cx43 phosphorylation *in vivo*. Based on the available data, it seems that PP1 is the main player during acute dephosphorylation of Cx43, as seen during cardiac ischemia, whereas PP2A may become the dominant player during long-term pathological alterations such as non-ischemic heart failure. The different results may, however, also be species dependent and further studies are needed before the final conclusion can be drawn.

### A PERSPECTIVE ON CONNEXIN43 PHOSPHORYLATION

As outlined above, phosphorylation of Cx43 has been a subject for intense investigation for more than three decades. These investigations have clarified that the regulation of Cx43 gap junction channels by phosphorylation is an extremely complex matter, which includes at least 21 different phosphorylation sites and a growing list of more than 10 different kinases and phosphatases. In addition, it is now clear that the different phosphorylation sites may interact and depend upon each other and site specific changes in phosphorylation may exert different effects on electric and metabolic coupling. Furthermore, it seems that the effects of altered Cx43 phosphorylation may be both species and cell type dependent. All of these considerations should be taken into account when planning future studies on Cx43 phosphorylation, as well as during the interpretation of such studies.

### PHOSPHORYLATION OF CONNEXIN26

For many years, it was generally thought that Cx26 was not a phospho-protein, because of its fairly short C-terminal tail, consisting of only 11 amino acids. A mass spectrometry study, however, showed that Cx26 is phosphorylated in the intracellular N-terminal tail at either position Asp2, Thr5, or Ser8 when expressed in HeLa cells (Locke et al., 2006). Importantly, the same phosphorylation was not detected in Cx26 from liver tissue, where the same amino residues were found to be hydroxylated instead (Locke et al., 2006). In a later study, Harris and Locke (2009) also found indications for potential phosphorylation sites in the cytoplasmic loop (Thr123) and the extracellular loop (E2; Thr177, Ser183 and Thr186) of Cx26 (Locke et al., 2009). Nevertheless, the physiological role of Cx26 phosphorylation remains unknown.

### PHOSPHORYLATION OF CONNEXIN32

As for Cx26, Cx32 also contains phosphorylation sites in the N-terminal domain, more specifically Thr4, Tyr7, Thr8, or Ser11, which was identified as phosphorylated in Cx32 from both HeLa cells and mouse liver (Locke et al., 2006). In addition, Cx32 from HeLa cells was phosphorylated in the C-terminal tail at position His237, Ser233, and/or Ser240 (Locke et al., 2006). In support of the findings by mass spectrometry, work on isolated hepatocytes have shown that both PKA and PKC may phosphorylate Ser233, whereas CaMKII may phosphorylate other serine and threonine sites in Cx32, but not Ser233 (Saez et al., 1990). Cx32 is also identified as a target for epidermal growth factor (EGF)-receptor tyrosine kinase (Díez et al., 1998). The specific tyrosine residue(s), which is/are targeted by EGF-receptor tyrosine kinase, was not revealed, but the study showed that binding of calmodulin to Cx32 prevented EGF-receptor tyrosine kinase induced phosphorylation. Based on this finding, it is likely that the target for EGF-receptor tyrosine kinase phosphorylation is located in one of the calmodulin binding sties in Cx32, which is amino acid no. 1–27 in the N-terminal tail and amino acid no. 216–230 located in the C-terminal tail (Török et al., 1997).

Further experimental studies are needed in order to reveal to physiological role of Cx32 phosphorylation in biological systems.

### PHOSPHORYLATION OF CONNEXIN40

Cx40 is also regulated by post-translational phosphorylation (Traub et al., 1994; van Rijen et al., 2000), but the specific sites are yet to be studied in detail. So far, it is known that both PKA and PKC are able to incorporate P^32^ into Cx40 in transfected human cells and that 8-Br-cAMP induced PKA activation causes an electrophoretic mobility shift of Cx40. Furthermore, van Rijen et al. (2000) showed that Cx40 gap junction channels increase both macroscopic conductance and metabolic coupling, when subjected to PKA-induced phosphorylation in SKHep1 cells.

Recently, it was also shown that reduced serine phosphorylation of Cx40 correlates with reduced electrical coupling between microvascular endothelial cells (EC) during sepsis (Bolon et al., 2008). This process was prevented by PKA activation and mimicked in control cells by PKA inhibition, which indicates that the involved phosphorylation site(s) is/are a target for PKA.

Since Cx40 is the predominant connexin found in the atria, it can be hypothesized that dephosphorylation of Cx40 and decreased electrical coupling in the atria may play a role in the patogenisis of atrial fibrillation. This, however, remains an interesting question for future research.

### PHOSPHORYLATION OF CONNEXIN45

Cx45 is expressed in many different tissues and the only member of the connexin family, whose absence is lethal in the embryonic state (Kruger et al., 2000; Kumai et al., 2000). Even so, the post-translational regulation of Cx45 has been less extensively studied compared to other connexins. Serine phsophorylation of Cx45 is found in both mouse kidney (Butterweck et al., 1994), cultured neonatal rat ventricular myocytes (Darrow et al., 1995), as well as in HeLa transfectants (Hertlein et al., 1998). The latter study found that exchange of the serine residues at position 374, 376, 378, 381, 382, 384, 385, 387, and 393 for other amino acids or deletion of this part of the carboxy-terminal led to an 89% decrease in the phosphorylation signal. This indicates that these nine serine residues are the main sites for Cx45 phopshorylation. They did, however, also find signals for phosphothreonine and phosphotyrosine after metabolically labeling with ^32^P-orthophosphate. Even when all the above mentioned carboxy-terminal serine residues of Cx45 were replaced, it did not interfere with intracellular trafficking or assembly of gap junction channels at the plasma membrane. Furthermore, HeLa cells transfected with the Cx45 mutant showed the same extend of dye transfer as cells transfected with wild type Cx45 (Hertlein et al., 1998). Instead, they found that mutation of the nine serine residues in the cytoplasmic tail caused an increased Cx45 degradation. When all nine serine residues or the double serine residues Ser381 – Ser382 or Ser384 – Ser385 were exchanged, the Cx45 half-life time was reduced with up to 50%.

A recent study based on tandem mass spectrometry showed that CaMKII and CK1 phosphorylates Cx45 *in vitro* (Bao et al., 2011). Specifically, CaMKII was found to phosphorylate serine 326, 381, 382, 384, 385, 387, and 393 along with threonine 337, whereas CK1 phosphorylate serine 326, 382, 384, 387, and 393. Another study, also based on HeLa cells transfected with Cx45, reported that activation of PKA and MAP kinase increases Cx45 phosphorylation when analyzed by western blotting (van Veen et al., 2000). PKA and MAP kinase induced Cx45 phosphorylation was associated with a decreased macroscopic junctional conductance, whereas activation of PKC was found to increase macroscopic conductance of Cx45 channels. However, the effect of PKC occurred in the absence in any apparent changes in Cx45 phosphorylation. Together, all of these studies show that Cx45 contains several phosphorylation sites, which are involved in the regulation of Cx45 degradation and macroscopic conductance, at least in HeLa cells. However, the role of Cx45 phosphorylation *in vivo* during both physiological and pathophysiological conditions remains an open question for future research.

### PHOSPHORYLATION OF CX46 AND CX50

Cx46 and Cx50 are known to combine and form heteromeric gap junction channels in ocular lens fibers and both connexins play an important role in lens growth and maintenance of lens transparency. Mass spectrometry analysis of Cx46 and Cx50 isolated from bovine lenses identified a total of 11 and 18 phosphorylation sites, respectively (Wang and Schey, 2009). All of the identified phosphorylation sites in Cx46 are located in the C-terminal tail, whereas three of the identified sites in Cx50 are located in the cytoplasmic loop. The physiological response to altered Cx46 and Cx50 phosphorylation is not yet fully understood. In chicken, however, caspase-3-like protease induced truncation of lens Cx45.6 (the chicken homolog of Cx50) is inhibited by casein kinase II (CKII) induced phosphorylation of Ser363 (Yin et al., 2001). Calpain mediated cleavage of lens Cx46 and Cx50 is proposed to occur naturally during the maturation of lens fibers (Lin et al., 1997; Yin et al., 2001), and the data by Yin et al. (2001) implies that Cx46 and Cx50 cleavage may be regulated by Cx46 and Cx50 phosphorylation.

## UBIQUITINATION

Ubiquitin is a 76 amino acid polypeptide that is found in almost all eukaryotic cells. Structurally, it consists of a globular domain, formed by a β-sheet and an α-helix, with the N- and C-termini protruding out. It is important for protein trafficking, in particular for labeling proteins for proteosomal destruction and recycling. Aaron Ciechanover, Avram Hershko, and Irwin Rose were awarded the Nobel Prize in Chemistry in 2004 for the discovery of ubiquitin-mediated protein degradation.

The enzymatic process of ubiquitin binding (ubiquitination or ubiquitinylation) to a target protein begins with activation of ubiquitin by an E1 ubiquitin activating enzyme that forms a bond between the carboxyl group of the C-terminal glycine residue (Gly 76) of ubiquitin and an E1 cysteine. This step is followed by transfer of ubiquitin from E1 to a cysteine residue of the ubiquitin-conjugating enzyme E2. Finally, the E2-ubiquitin conjugated enzyme associates with an E3 ubiquitin-protein ligase (as reviewed by Schulman and Harper, 2009; Weissman et al., 2011). The E3 enzymes function as substrate recognition module and facilitates the formation of a bond between a lysine of the target protein and the C-terminal glycine of ubiquitin. In most organisms, there is only one E1, several different E2 and hundreds of E3s. By determining the timing and the substrate of the ubiquitination process, the E3 ubiquitin-protein ligases are the central regulatory determinants of the ubiquitination process. The E3 ubiquitin-protein ligases can be classified into (1) The HECT (homologous to E6-AP carboxy terminal) E3s (Huibregtse et al., 1995), (2) The RING (really interesting new gene) E3s (Lorick et al., 1999) or the closely related U-box E3s.

Homologous to E6-AP carboxy terminal E3 participates directly in ubiquitination by forming a bond with ubiquitin prior to the transfer of ubiquitin to the target protein. HECT E3s contains 3 WW domains, WW1–3. WW domains are short domains contain 38 to 40 amino acid residues, including two conserved tryptophan residues, hence the name WW domain. The domain form a triple-stranded β sheet that binds proteins with particular proline-motifs (PY motifs, XXPPXY, where P is a proline, X is any amino acid and Y is tyrosine) and/or phosphoserine and phosphothreonin containing motifs and thereby mediate target recognition (Nguyen et al., 1998).

An ubiquitin molecule contains a total of seven lysine residues. Following addition of a single ubiquitin to a protein (monoubiquitination), further ubiquitin molecules can be conjugated to lysine residues in the bound ubiquitin, resulting in a polyubiquitin chain. The first identified type of polyubiquitin chains were linked via lysine 48 (Lys48 forming an isopeptide bond to Gly76), however, a wide variety of linkages involving all possible lysine residues (Lys6, 11, 27, 29, 33 and 63) have been demonstrated, reviewed by (Ikeda and Dikic, 2008; Kulathu and Komander, 2012). Further, some substrates contain multiple lysine residues that can be modified by addition of ubiquitin molecules or ubiquitin chains (multiubiquitination).

The various types of ubiquitin modifications have been linked to distinct physiological functions in cells. Multi-monoubiquitination is associated with DNA repair and receptor endocytosis, and lysosomal degradation or recycling to the surface (Dikic et al., 2009) whereas lysine 48-linked polyubiquitin chains label proteins for proteasomal degradation (Hershko and Ciechanover, 1998). Homotypic ubiquitin chains (e.g., chains consisting of only Lys6, 11, 27, or 29 linkages) are also found, and may be involved in different cellular processes such as signaling, trafficking, DNA damage response, cell cycle regulation or endoplasmic reticulum associated degradation (ERAD) as reviewed by (Komander, 2009).

### UBIQUITINATION OF CONNEXINS

Ubiquitination may be involved in several important steps in the life cycle of connexins (reviewed by Kjenseth et al., 2010 and Su and Lau, 2012). The newly synthesized connexins undergo quality control in the endoplasmic reticulum and polyubiquitination targets misfolded connexin proteins to ERAD and proteasomal degradation (VanSlyke and Musil, 2002). CIP75 (connexin interacting protein 75) facilitates this process (Li et al., 2008). After insertion into gap junction plagues in the membrane, ubiquitination by the E3 ubiquitin ligase Nedd4 is thought to target connexins for endocytosis and lysosomal degradation (Leykauf et al., 2006). The connexins remain ubiquitinated throughout the internalization process that is assisted by EGF receptor pathway substrate 15 (Esp15; Girao et al., 2009). In the early endosomes, the ubiquitin binding proteins Hrs (hepatocyte growth factor-regulated tyrosine kinase substrate) and Tsg101 (tumor susceptibility gene product 101) interacts with ubiquitinated connexins and determines whether the connexins are targeted for recycling or for deubiquitination followed by degradation (Leithe et al., 2009). Connexins targeted for destruction may follow several alternative endocytotic pathways, however, there is increasing evidence suggesting that the final destination is lysosomal degradation (Laing et al., 1997; Musil et al., 2000; Girao and Pereira, 2003; Rivedal and Leithe, 2005; Piehl et al., 2007; Falk et al., 2012).

### ERAD AND CIP75

Approximately 40% of newly synthesized Cx43 and Cx32 may undergo ERAD (VanSlyke and Musil, 2002). The level of ERAD is strongly dependent on cellular stress. Oxidative and thermal stress reduce ERAD and more connexins reach the membrane and form functional gap junctions (VanSlyke and Musil, 2002). The mechanism behind cellular stress-induced ERAD is not known (VanSlyke and Musil, 2002). Interestingly, a Cx32 mutation, where Glu 208 is replaced by a lysine from a patient with X-linked peripheral neuropathy Charcot-Marie-Tooth was found to cause nearly a 100% of the newly synthesized Cx32 to undergo ERAD (VanSlyke et al., 2000; Kelly et al., 2007), however, under cellular stress, the polyubiquitination of this Cx32 mutation is reduced. The mutated proteins accumulate in the ER and may contribute to the disease by co-assembling with wild type connexin (VanSlyke et al., 2000; Kelly et al., 2007).

The CIP75 protein is involved in ERAD by translocating the ubiquitinated connexins across the endoplasmatic reticulum membrane as well as bringing the connexins targeted for degradation to the proteasomes during the ERAD process (Li et al., 2008). The Cx43 C-terminal harbors a proline-rich area corresponding to the PY motif (Thomas et al., 2003a), that is important for E3 ubiquitin-ligase target recognition (Nguyen et al., 1998). The interaction between CIP75 and Cx43, takes place between a C-terminal ubiquitin-associated domain of CIP75 and this PY motif, as well as multiple phosphorylation sites located between Lys264 and Asn302 of Cx43 (Li et al., 2008). CIP75 contains domains that interact with the proteasomal complex and overexpression experiments suggested that CIP75 stimulates proteasomal degradation of Cx43 (Li et al., 2008). CIP75 can bind free mono-ubiquitin and Lys48-linked ubiquitin chains *in vitro* as well as ubiquitinated proteins in cellular lysates (Su et al., 2010). However, it was demonstrated that Cx43 associated with CIP75 is not ubiquitinated, and a mutant form of Cx43 lacking lysine residues and thus incapable of ubiquitination retained the capacity to interact with CIP75. This suggests that although CIP75 can interact with ubiquitinated proteins, its interaction with Cx43 and stimulation of Cx43 proteasomal degradation does not necessarily require ubiquitination (Su et al., 2010).

### UBIQUTINATION AND INTERNALIZATION OF CONNEXINS IN GAP JUNCTIONAL PLAQUES

Laing and Beyer (1995) were the first to describe ubiquitination of Cx43 and ubiquitin-mediated proteasomal degradation. Proteasomal inhibition and inactivation of the E1 ubiquitin activating enzyme resulted in stabilization of Cx43 at the plasma membrane. It was later found that 50% of Cx43 plaques are ubiquitinated, however, ubiquitination is most predominant in older plagues, suggesting that ubiquitination may signal endocytosis of older gap junction from the plasma membrane to the lysosomes for degradation (Laing et al., 1997; Musil et al., 2000; Girao and Pereira, 2003; Rivedal and Leithe, 2005). The role of the proteasomes in endocytosis and degradation of connexins from gap junctional plaques is not clear. Several studies have demonstrated that endocytosis of Cx43 is repressed in the presence of proteasomal inhibitors, suggesting that there may be an intricate interplay between lysosomes and proteasomes as reviewed in (Kjenseth et al., 2010; Falk et al., 2012).

The E3 ubiquitin ligase Nedd4 (neural precursor cell expressed, developmentally downregulated 4) was the first E3 ubiquitin ligase to be shown to interact with connexin (Leykauf et al., 2006). Nedd4 is thought to play an important role in the regulation of connexins in gap junction plaques and ubiquitination by Nedd4 targets connexins for internalization (Leykauf et al., 2006; Girao et al., 2009).

Nedd4 belongs to the HECT family of E3 ubiquitin ligases (Huibregtse et al., 1995) and contains three WW domains. The interaction between Nedd4 and Cx43 takes place between the PY motif of the Cx43 C-terminus (Leykauf et al., 2006; Girao et al., 2009) and the three WW domains of Nedd4 (Leykauf et al., 2006). WW1 and WW2 domains mainly interact with the unphosphorylated form of Cx43, whereas WW3 binds phosphorylated and unphosphorylated forms equally (Leykauf et al., 2006). Only the WW2 domain binds to the PY motif (Leykauf et al., 2006). Other groups have demonstrated interaction of Cx43 with additional E3 ligases, including the other members of the HECT family smad ubiquitination regulatory factor-2 (Smurf2; Fykerud et al., 2012) and WWP1 as well as the RING E3 ligase Tripartite motif-containing protein 2 (TRIM21) as reviewed by Su and Lau (2012).

### EPIDERMAL GROWTH FACTOR MAY STIMULATE UBIQUINATION OF CONNEXINS IN GAP JUNCTIONAL PLAQUES

Many growth factors, such as EGF and tumor promotors, including TPA inhibits intercellular communication by inducing Cx43 ubiquination followed by endocytosis and degradation (Leithe and Rivedal, 2004a; Leithe et al., 2009; Sirnes et al., 2009). In rat liver epithelial cell lines, application of EGF activates the MAP kinase pathway resulting in Cx43 hyperphosphorylation (Lau et al., 1992; Kanemitsu and Lau, 1993; Leithe and Rivedal, 2004a) on Ser255, 279 and 282 in the C-terminus of Cx43 (Warn-Cramer et al., 1996, 1998). The EGF mediated hyper-phosphorylation of Cx43 was followed by ubiquitination resulting in a rapid transient decrease in intracellular coupling (Leithe and Rivedal, 2004a). However, EGF does not only uncouple gap junctions, they also induce disorganization, endocytosis and degradation of Cx43 plaques (Leithe and Rivedal, 2004a).

Besides stimulating the MAP kinase pathway, EGF activates phospholipase C, resulting in hydrolysis of phosphatidylinositol 4,5-bisphosphate (PIP2) into inositol 1,4,5-trisphosphate (IP3) and diacylglycerol (DAG; van Rheenen et al., 2007). Reduced PIP2 levels inhibit gap junctional coupling in both fibroblasts (van Zeijl et al., 2007) and cardiomyocytes (Hofgaard et al., 2008). Stimulation of cultured cardiomyocytes from neonatal rats with noradrenaline or angiotensin II decrease cell coupling and action potential conduction velocity mediated via a decrease in PIP2 levels (Hofgaard et al., 2008) and in a later study it was demonstrated that noradrenaline induced uncoupling was associated with ubiquitination of Cx43, possibly via Nedd4, and subsequent internalization of Cx43 (Mollerup et al., 2011).

The tumor promoting phobol ester TPA activates the MAP kinase pathway, as well as PKC (Leithe and Rivedal, 2004a; Sirnes et al., 2009). This in turn induce hyperphosphorylation of Cx43 followed by monoubiquitination at multiple sites (Leithe and Rivedal, 2004b; Leithe et al., 2006). Eps15 is recruited to the membrane to facilitate endocytosis of Cx43 (Girao et al., 2009). Ubiquitination may also be important for the transport from the endosome to the lysosomes, a process facilitated by Hrs and tsg101 (Leithe et al., 2009).

Recently, the role of ubiquitination in regulation of Cx43 gap junction turnover was questioned in a study by Dunn et al. (2012). They demonstrated that a Cx43 construct, where all lysine residues were replaced by arginine residues, behaved similarly to wild-type (Dunn et al., 2012). This is in line with the failure to identify a specific lysine acceptor for ubiquitination on Cx43 and may imply that ubiquitination of Cx43 is not crucial for regulation of Cx43 gap junction turn over. As Cx43 localization is regulated by phosphorylation, the authors hypothesized that another ubiquitinated protein may regulate Cx43 retention or degradation. Since phosphorylation is crucial for localization and activity of Cx43, the effect of inhibitors of the various kinases know to affect Cx43 was tested. These experiments suggests that Akt activity controls gap junction stability through phosphorylation (Dunn et al., 2012). When Akt is activated by phosphorylation it translocates to the plasma membrane, where it phosphorylates membrane proteins, inclusive Cx43 on Ser373 and Ser369 (Park et al., 2007), resulting in stabilization of gap junction plaques (Dunn et al., 2012). Interestingly, Akt is a target for ubiquitination and proteasomal degradation (Yang et al., 2009), which may potentially explain the effect of proteasomal and lysosomal inhibitors on connexin expression.

## SUMOYLATION

Small ubiquitin-like modifier (SUMO) proteins are a small family of proteins that are structurally and functionally related to ubiquitin. In humans, the SUMO protein family consists of 3 members (SUMO1-3). SUMO 2 and 3 show a high degree of similarity (Saitoh and Hinchey, 2000). The structural folding of SUMO proteins is similar to that of ubiquitin, but there is little overlap in the amino acid sequence. The consensus motif for SUMOylation is ΨKXD/E where Ψ is a large hydrophobic residue, K is the acceptor lysine, X is any amino acid followed by an acidic residue (Rodriguez et al., 2001)

Analog to ubiquitination, SUMOylation is controlled by an enzymatic cascade resulting in covalently attachment of SUMO to lysine residues in the target protein (reviewed by Hay, 2005 and Gareau and Lima, 2010). A C-terminal peptide is cleaved from SUMO by sentrin-specific proteases (SENPs) to reveal a di-glycine motif. SUMO then becomes bound to an E1 enzyme (or SUMO activating enzyme, SAE). It is then passed to an E2, which is a conjugating enzyme, Ubc9, which is able to directly recognize substrates with a SUMOylation consensus motif (reviewed by Hay, 2005). However, the SUMO moiety can also be transferred to one of a small number of E3 ligating proteins that conjugates SUMO to target proteins. While in ubiquitination an E3 ligating protein is essential for the process, evidence suggests that the E2 is sufficient in SUMOylation, as long as the consensus sequence is present. The SUMO moieties can be removed by SENPs that serve dual functions in the SUMOylation circle (Gareau and Lima, 2010). SUMO2 and SUMO3 contains the SUMOylation consensus motif, which can be utilized to form poly-SUMOylation chains on the target protein (reviewed by Hay, 2005; Gareau and Lima, 2010).

In contrast to ubiquitination, SUMOylation is not used to target proteins for degradation but is rather involved in the regulation of various cellular processes, including transcriptional regulation, protein targeting and stability, response to stress, progression through cell cycle and apoptosis (Geiss-Friedlander and Melchior, 2007).

### CONNEXINS AND SUMOYLATION

Connexins was first described to be regulated by SUMOylation by Kjenseth et al. (2012). In transfected HeLa cells, all three members of the SUMO family increased Cx43 expression and gap junction formation. SUMO2-3 had the strongest effect, resulting in a doubling of Cx43 expression and for Cx43 coexpressed with SUMO3, an increased intracellular communication was demonstrated (Kjenseth et al., 2012).

As previously described, the Cx43 N-terminus contains 3 lysine residues, the intracellular loop 11 and the C-terminus contains 9 residues, but none of these are found in a SUMOylation consensus motif. However, mutational scanning revealed that SUMO1-3 conjugated SUMO groups to Lys144 found in the intracellular loop and Lys237 in the proximal C-terminus of Cx43 (Kjenseth et al., 2012). Lys144 and 237 are evolutionary conserved in Cx43, and interestingly, Lys144 is also conserved in 8 out of 20 human connexins isoforms, including Cx26 and Cx32 (Kjenseth et al., 2012) suggesting that other connexins may also be regulated by SUMOylation. Directly downstream of Lys144 is a large hydrophobic residue and thus this region may function as an inverted SUMOylation consensus motif as described (Matic et al., 2010). Cx43 proteins, where Lys144 or 237 were replaced by arginine residues, had reduced Cx43 gap junction formation and reduced protein levels (Kjenseth et al., 2012). In addition to lysine 144 and 237, other lysines may be SUMOylated.

For SUMO1 or SUMO2, Cx43 was conjugated to single moieties, whereas for SUMO 3 multiple proteins could be conjugated to Cx43, either as multiple mono-SUMOylation or as poly-SUMOylation (Kjenseth et al., 2012). The majority of the SUMOylated Cx43 was found in the Triton X-100 soluble fraction (Kjenseth et al., 2012), suggesting that Cx43 were not organized in functional gap junctions as gap junction plaques are Triton X-100 insoluble (Musil and Goodenough, 1991; VanSlyke and Musil, 2000). However, gap junctions seem to aquire Triton solubility before internalization (Sirnes et al., 2008) and work in cultured cardiomyocytes show that inhibition of the proteasome and lysosome increase both Triton solubility of Cx43 and intercellular coupling simultaneously, indicating that communicating gap junctions can be part of the soluble fraction (Mollerup et al., 2011).

The molecular mechanism behind the SUMOylation mediated increase in Cx43 protein level is not presently known. It is also unknown, in which subcellular compartment SUMOylation takes place and further studies are needed to determine whether SUMOylation affects intracellular Cx43 trafficking (Kjenseth et al., 2012).

## NITROSYLATION

Recent evidence suggests that *S*-nitrosylation of cysteine residues may be an important post-translational modification of connexins (Retamal et al., 2006, 2009; Straub et al., 2011). *S*-nitrosylation is the reversible, covalent addition of NO to the thiol side chain of cysteine and nitrosylation appears to be the major mechanism through which NO exerts its effects on cellular function (Hess et al., 2005; Anand and Stamler, 2012; Hess and Stamler, 2012). The degree of nitrosylation is subject to dynamic regulation through the concerted activity of multiple nitrosylases and denitrosylases, i.e., enzymes that adds or removes NO groups to/from proteins (Hess and Stamler, 2012). Although many proteins contain multiple cysteins, and therefore have many potential sites for nitrosylation, it appears as if only a few of the potential sites are actually subject to nitrolysation, and consequently, that the effects on protein function results from the modification of only one or a few –SH groups in a given protein (Hess and Stamler, 2012). Nitrosylation may directly influence protein function, but interestingly may also have an indirect effect through modification of other post-translational modifications, e.g., phosphorylation, acetylation or ubiquitination (Hess and Stamler, 2012).

### NITROSYLATION OF CONNEXINS

NO has been shown to affect both gap junctions and hemichannels, but only a few studies provides direct evidence showing that the effect is correlated to changes in nitrosylation of the connexins. In the vascular wall the myoendothelial junctions (MEJs) plays an important role in coordinating the activity of EC and vascular smooth muscle cells (VSMC; Sandow et al., 2012). The MEJs form where cellular extensions from the VSMC meet the EC, and heterotypic junctions are formed at the site of contact between the plasma membranes from the two cell types. In the VSMC, the major connexins present are Cx43 and Cx45, whereas Cx37 and Cx40 are found in the EC (Sandow et al., 2012). Interestingly, the MEJ also appears to have a high content of eNOS coexpressed with the gap junction plaques. Cx43 was found to be constitutively nitrosylated in the MEJ, and denitrosylation induced by addition of phenylephrine resulted in a reduced movement of inositol triphosphate from the VSMC to the EC via the MEJ gap junctions, and this decreased gap junction permeability was associated with an decreased nitrosylation of Cx43 at the Cys271 residue in the intracellular C-terminal part of the protein (Straub et al., 2011). Other data also support the notion that nitrosylation may play role in modifying gap junctions in the vasculature. Two independent studies show that NO donors decrease the permeability between ECs toward small molecules and reduce the electrical coupling of gap junctions composed of Cx37 (Kameritsch et al., 2005; McKinnon et al., 2009). In both cases the effect was independent of the action of NO on cGMP, but none of the studies provided any direct evidence of changes in nitrosylation of the involved proteins. Taken together the studies suggest that nitrosylation of connexins may play an important role in regulation of the intercellular communication in the microcirculation. Intercellular communication is a prerequisite for conducted vasomotor responses, and several conditions like hyperglycemia, hypertension and sepsis is associated with changes in both vascular conduction and NO production, however there is at present no information linking these changes to changes in nitrosylation of the vascular connexins (Gustafsson and Holstein-Rathlou, 1999; McKinnon et al., 2006, 2009; Rai et al., 2008; Rodrigo et al., 2011; Sudano et al., 2011).

Nitrosylation may also play a role in the regulation of hemichannel function. Ischemia and/or hypoxia are associated with an increased production of NO, and at the same time an increase in Cx43 and Cx46 hemichannel permeability has been observed (Retamal et al., 2006, 2009). The increased hemichannel permeability leads to the loss of intracellular ions and small organic compounds, which may contribute to cell death. Retamal et al. (2006) showed that metabolic inhibition of cortical astrocytes resulted in an increased cell permeability as determined by dye uptake. The increased dye uptake was associated with both a dephosphorylation and a nitrosylation of the Cx43 hemichannels. Addition of reducing agents, which decreases nitrosylation, reduced the cellular permeability without having any effects on the phosphorylation level. Since addition of external NO donors also increases cell permeability in the same system, it was concluded that *S*-nitrosylation play an important role in regulating hemichannel permeability in astrocytes (Retamal et al., 2006). The same group showed that addition of the NO-donor GSNO to Cx46 hemichannels expressed in *Xenopus* oocytes caused an increased voltage sensitivity and current amplitude (Retamal et al., 2009). On the other hand, treatment of the cells by reducing agents reversed the effect of GSNO on hemichannel currents, and mutation of the two cysteine residues in the carboxy-terminal part of Cx46 abolished the effect of GSNO.

## METHYLATION AND ACETYLATION

Although methylation and acetylation are mainly known for their epigenetic regulation of gene transcription by DNA methylation and histone acetylation, there is emerging evidence that connexins may be directly modified. By mass spectrometry, Cx26 was shown to be both methylated and acetylated (Locke et al., 2009). Five methylated sites were detected, one of which (Arg75) is associated with deafness causing mutations (R75W and R75Q), indicating that methylation of this site plays an important physical role. Several studies show that Cx26 channels carrying the R75W mutation are dysfunctional, and although the channels are trafficked to the membrane, they do not result in electrical or metabolic coupling (Richard et al., 1998; Marziano et al., 2003; Oshima et al., 2003; Thomas et al., 2003b; Chen et al., 2005). The mutant channels even had a dominant negative effect on not only wild type Cx26 (Richard et al., 1998; Marziano et al., 2003; Oshima et al., 2003) but also on Cx30 (Forge et al., 2003; Marziano et al., 2003) with which it coexpresses in the inner ear. Besides disrupting cell to cell communication, mutation of Arg75 also reduced the permeability and macroscopic conductance of Cx26 hemichannels, as well as altered their voltage dependence (Chen et al., 2005; Deng et al., 2006).

Besides methylation, Cx26 is also acetylated on six sites including Lys15 and Lys102 for which disease causing mutations/deletions are known (K15T, del102K; Locke et al., 2009). Acetylation is also described in bovine Cx49 (ortholog of human Cx50) on the N-terminal arginine, a commonly occurring modification (Shearer et al., 2008), which was also reported for Cx26 (Locke et al., 2009). An important physiological role for acetylation was also reported for Cx43 in mdx mice, a model of Duchenne muscle dystrophy (Colussi et al., 2011). Cx43 is acetylated and lateralized in mdx mice and interference with acetylases/deacetylases indicated causality between the two. Furthermore, mutagenesis of three lysines (9, 234 and 264), predicted as acetylation targets, showed that acetyl-mimetic mutations led to intracellular localization, whereas Cx43 with un-acetylatable mutations were resistant to acetylating conditions (Colussi et al., 2011).

The data from Cx26 and Cx43 clearly shows that methylation and acetylation will likely emerge as important PTMs in the regulation of intercellular communication mediated by these and other connexins.

## GLUTAMATE γ-CARBOXYLATION

The conversion of glutamic acid to γ-carboxyglutamic acid is a vitamin K dependent PTM, which was originally described for blood clotting proteins (Nelsestuen et al., 1974; Stenflo et al., 1974). The mass spectrometry study by Harris and Locke (2009) identified glutamate γ-carboxylations in Glu42 and Glu47 in the extracellular loop (E1), as well as Glu114 in the cytoplasmic loop of Cx26 (Locke et al., 2009). γ-carboxylation is an irreversible modification, which generates a high-affinity Ca^2^^+^ binding site. This indicates that γ-carboxylation of Cx26 may be involved in the regulation of Cx26 Ca^2^^+^ sensitivity and, in this context, it is noteworthy that mutations at Glu42 and Glu47 cause Cx26 dependent deaf mutations (Lee and White, 2009). This implies that glutamate γ-carboxylation of Cx26 may play a physiological role and future studies may reveal if other Ca^2^^+^ sensitive connexins, such as Cx43 and Cx50, are also subject to γ-carboxylation.

## CONCLUSION

The literature clearly shows the importance of PTMs in modifying gap junctional coupling and dependent on the connexin and the PTM type, may either increase or decrease intercellular coupling. This is achieved by regulating connexin function at all levels, including oligomerization, trafficking, channel activity and connexin degradation. The importance of these phenomena is underpinned by the fact that several disease causing mutations affect amino acids known to be PTM sites, as described above.

The large and increasing number of PTMs that regulate connexins comprises a major challenge in unraveling their function and importance. For instance, Cx43 contains at least 24 PTM sites and the number of combinations thereof is overwhelming and their investigation beyond the reach of current methods for studying connexins. Most studies address only one or a few modifications at a time using site directed phosphospecific antibodies or mutagenesis. Such approaches carry the risk of overlooking confounding influence of other modifications and may explain some of the contradicting results in the literature. The field of proteomics is rapidly evolving and may eventually disclose quantitative important PTMs and combinations thereof occurring in physiology and pathophysiology. At present, the published mass spectrometry studies of connexins are limited to determinations of whether a certain modification is detectable or not. The threshold for such detection is undefined and not necessarily linked to the threshold for achieving a significant physiological effect although this is often implied. In some cases, such as for Ser306 in Cx43, one may find a change in phosphorylation that passes the threshold for detection after some intervention, in this case ischemia (Axelsen et al., 2006), and be lucky enough that site phosphorylation is associated with a change in channel function (Procida et al., 2009). The importance of other sites could pass undetected simply because they did not pass threshold. Quantitative proteomics are coming to our rescue and rapidly evolving (Cox and Mann, 2011), but even these techniques may not reveal coupling of PTMs at the single molecule level. In any case, the role of PTMs in regulating intercellular communication, will keep researchers busy for years to come.

## Conflict of Interest Statement

The authors declare that the research was conducted in the absence of any commercial or financial relationships that could be construed as a potential conflict of interest.
